# Thyroarytenoid muscle fiber orientation regulates the ability to modulate vocal fold vertical thickness

**DOI:** 10.1371/journal.pone.0337735

**Published:** 2025-12-04

**Authors:** Tsukasa Yoshinaga, Zhaoyan Zhang

**Affiliations:** 1 Department of Head and Neck Surgery, University of California, Los Angeles, California, United States of America; 2 Graduate School of Engineering Science, Osaka Unviersity, Toyonaka, Osaka, Japan; Tokai University, School of Medicine, JAPAN

## Abstract

While all speakers modulate their voice quality, some speakers do so more easily than others. Previous studies suggest that the ability to modulate voice quality depends on the ability to control vocal fold medial surface vertical thickness, among other factors. While it is well known that vocal fold vertical thickness can be increased by an inferior medial bulging of the vocal folds due to actions of the thyroarytenoid (TA) muscle, the underlying mechanism of how TA muscle activation produces inferior medial bulging remains unclear. In this computational study, we show that the TA muscle fiber orientation had a large effect on the extent of inferior medial bulging and vertical thickness increase under TA muscle contraction in a three-dimensional model of laryngeal muscle activation based on high-resolution magnetic resonance imaging. Maximum inferior medial bulging and thickness increase occurred when the TA muscle fiber orientation angle ranged between 22–32° relative to the anterior-posterior direction. At these optimal fiber angles, TA muscle activation was able to induce a horizontal rotation of the arytenoid cartilage, which medialized the vocal process and the inferior portion of the medial surface more than the superior portion of the medial surface, thus increasing vertical thickness. In contrast, the lateral cricoarytenoid muscle induced a rocking rotation of the arytenoid cartilage, which medialized the superior medial surface slightly more than the inferior medial surface, and thus had an opposite and much-reduced effect on vocal fold thickness. It is hypothesized that potential individual differences in TA muscle fiber orientation may contribute to individual differences in the ability to modulate voice quality.

## Introduction

The human voice is produced by a complex interplay between airflow and the vocal folds within the larynx [1,2]. Subtle changes in vocal fold posturing, including shape, tension, stiffness, and position, often lead to significant changes in voice quality. In particular, the vertical thickness of the vocal fold medial surface has been shown to be a key determinant of voice quality [3,4]. Changes in vertical thickness influence the glottal closure pattern and the resulting voice source characteristics [5]. When the vocal fold is too thin, incomplete glottal closure leads to high airflow and a smooth airflow waveform, with the voice spectrum dominated by a strong first harmonic and weak high-frequency harmonics. In contrast, thick vocal folds tend to produce prolonged glottal closure, low airflow, strong high-frequency harmonics, and, in some cases, irregular vibration and increased risk of vocal fold injury. The importance of medial surface thickness to vocal control has also been reported in recent experimental [6] and computational studies [7–9], and is well appreciated in the vocal pedagogy literature [e.g., 3,10,11].

**Fig 1:**
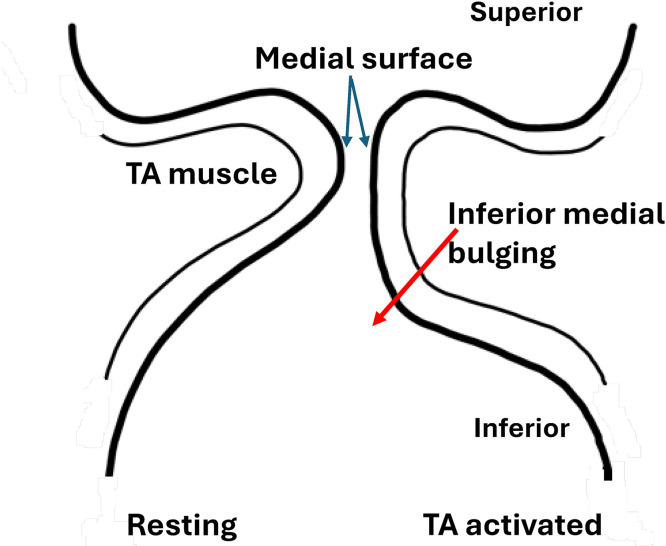
Inferior medial bulging of the vocal folds in coronal view. Activation of the thyroarytenoid (TA) muscle causes the inferior portion of the medial surface to bulge medially (red arrow) more than the superior portion of the medial surface, which increases the vertical thickness of the vocal fold medial surface.

The medial surface vertical thickness is regulated in humans by the intrinsic and extrinsic laryngeal muscles [4,10], among which the thyroarytenoid (TA) muscle has the largest effect. The TA muscle forms the bulk of the vocal folds. Its activation induces medial bulging of the vocal folds, particularly in the inferior portion of the medial surface, thus increasing the medial surface vertical thickness (Fig 1), which is well documented in the literature [1,2,4,11–16]. Despite this importance, it remains unknown what anatomical and physiological parameters determine the degree of inferior medial bulging under TA muscle activation. In fact, it is not clear why TA muscle activation induces medial bulging mostly in the inferior portion of the medial surface (Fig 1), which allows effective regulation of the medial surface vertical thickness, rather than a more uniform vocal fold bulging across the entire medial surface, which would not be as effective in regulating the medial surface vertical thickness.

The goal of this study was to clarify the biomechanical mechanisms of vocal fold inferior medial bulging due to TA muscle activation, using a computational model of human laryngeal muscle activation based on magnetic resonance images (MRI) of a male and a female larynx. Although previous computational studies have explored the effects of vocal fold posturing on phonation [17–26], the precise mechanisms by which TA muscle activation regulates inferior medial building and medial surface thickness have not been investigated. By systematically altering laryngeal muscle properties and boundary conditions, we will show that the orientation of TA muscle fiber has an important role in determining the degree of inferior medial bulging and regulating vocal fold vertical thickness. This suggests that individual anatomic differences in TA muscle fiber orientation may be an important contributing factor to individual differences in the ability to regulate vocal fold vertical thickness and voice quality, and thus need to be quantified in a large human population.

## Methods

### Laryngeal geometry reconstruction from MRI

Two excised human hemi-larynges (a 57-year-old male and an 82-year-old female) were obtained from autopsy and were used in MRI scanning. Since no human participants were involved and no identifying information was obtained from the excised larynges, no ethical approval was required, per the Institutional Review Board of the University of California, Los Angeles. The larynges were screened to confirm the absence of laryngeal pathology or intubation-related injury. Each larynx was placed in a cylindrical plastic container (5 cm in diameter) and scanned in a Bruker BioSpec 7 Tesla MRI (Bruker Biospin GmbH, Rheinstetten, Germany) with a 30-mm inner diameter surface coil. The spatial resolution was 0.1 × 0.1 × 0.1 mm^3^ [27].

**Fig 2:**
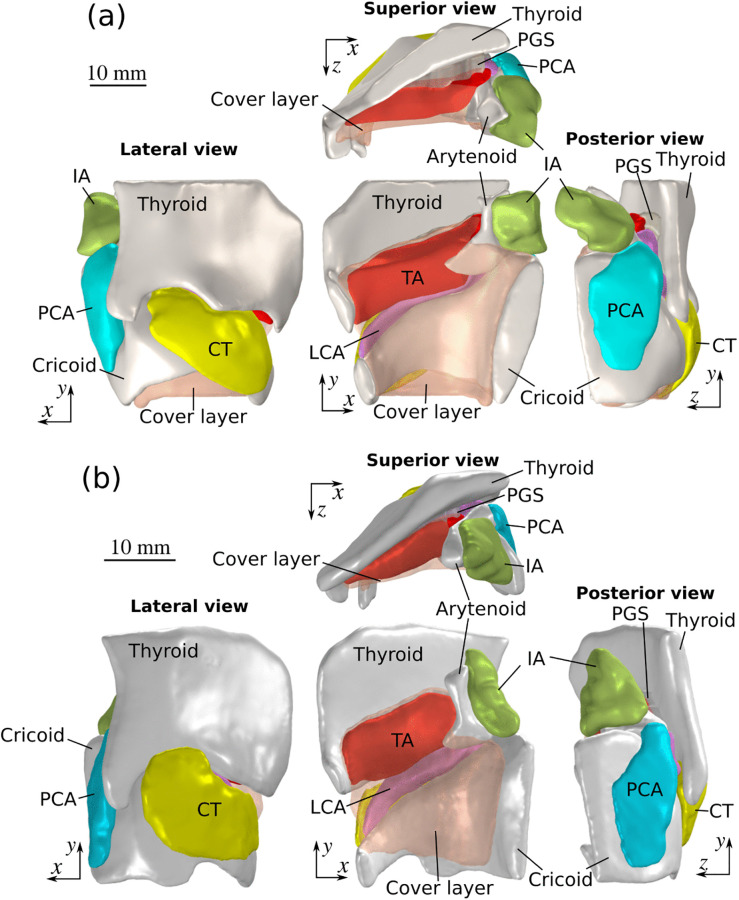
MRI-derived laryngeal geometry of (a) a 57-year-old male hemi-larynx and (b) an 82-year-old female hemi-larynx. TA: thyroarytenoid muscle; CT: cricothyroid muscle; LCA: lateral cricoarytenoid muscle; IA: interarytenoid muscle; PCA: posterior cricoarytenoid muscle; PGS: paraglottic space tissue located between the TA muscle and thyroid cartilage.

The three-dimensional laryngeal geometry, including the lamina propria (the cover layer of the vocal folds), laryngeal muscles (thyroarytenoid [TA], lateral cricoarytenoid [LCA], posterior cricoarytenoid [PCA], cricothyroid [CT], and inter-arytenoid [IA]), and the arytenoid, cricoid, and thyroid cartilages, was segmented using commercial software (Simpleware, Synopsys, Inc.) from the MRI images and reconstructed as shown in Fig 2. The paraglottic space (PGS), a soft tissue layer forming the posterolateral boundary between the vocal folds and thyroid cartilage, was also segmented and included in the model. The male vocal fold length was approximately 20 mm compared to 15 mm for the female vocal fold. The angle between the two thyroid laminae was greater in the male larynx than that in the female larynx. These are consistent with the typical morphological differences between males and females [1,2,28].

### Muscle and soft tissue modeling

Vocal fold deformation under muscle activation was obtained by solving the static equilibrium equation of the vocal folds,


$$\nabla \cdot {\left(𝐅\cdot 𝐒\right)}^{\text{T}}=\text{0}$$ (1)

where **S** is the second Piola–Kirchhoff stress tensor, and $\;𝐅=𝐈+\nabla {𝐮}^{\text{T}}$ is the deformation gradient tensor, with **u** being the displacement vector and **I** the identify tensor. The stress-strain relation of the vocal folds was defined using a strain energy function:


$$𝐒=\frac{\partial W}{\partial 𝐄}$$ (2)

where W is the strain energy function and 𝐄 is the Lagrangian strain tensor. For muscles, the strain energy function W includes both a passive component $W_{\text{passive}}$ and an active component $W_{\text{active}}$ associated with muscle activation,


$$W=W_{\text{passive}}+W_{\text{active}}.$$ (3)

The vocal fold (cover layer and the passive TA muscle) was assumed to be a nearly incompressible anisotropic hyperelastic material, modeled using the Holzapfel-Gasser-Ogden (HGO) formulation [29]:


$$W_{\text{passive}}=c\left({\overset{―}{I}}_{\text{1}}-\text{3}\right)+\frac{k_{\text{1}}}{\text{2}k_{\text{2}}}\left\{exp\left[k_{\text{2}}{\left({\overset{―}{I}}_{\text{4}}-\text{1}\right)}^{\text{2}}\right]-\text{1}\right\}+\frac{\kappa }{\text{2}}(J-\text{1}{)}^{\text{2}},$$ (4)

where ${\overset{―}{I}}_{\text{1}}$ is the first reduced invariant of the right Cauchy-Green tensor **C**, ${\overset{―}{I}}_{\text{4}}$ is the pseudo-invariant of **C** and the longitudinal direction **a**_vf_ (${\overset{―}{I}}_{\text{4}}={𝐚}_{vf}\cdot 𝐂{𝐚}_{vf}$), c is the material constant for the isotropic term, and $k_{\text{1}}$ and $k_{\text{2}}$ are the material constants for the anisotropic term. J is the Jacobian or the volume ratio between the deformed and undeformed tissues, and κ represents the bulk modulus.

**Fig 3:**
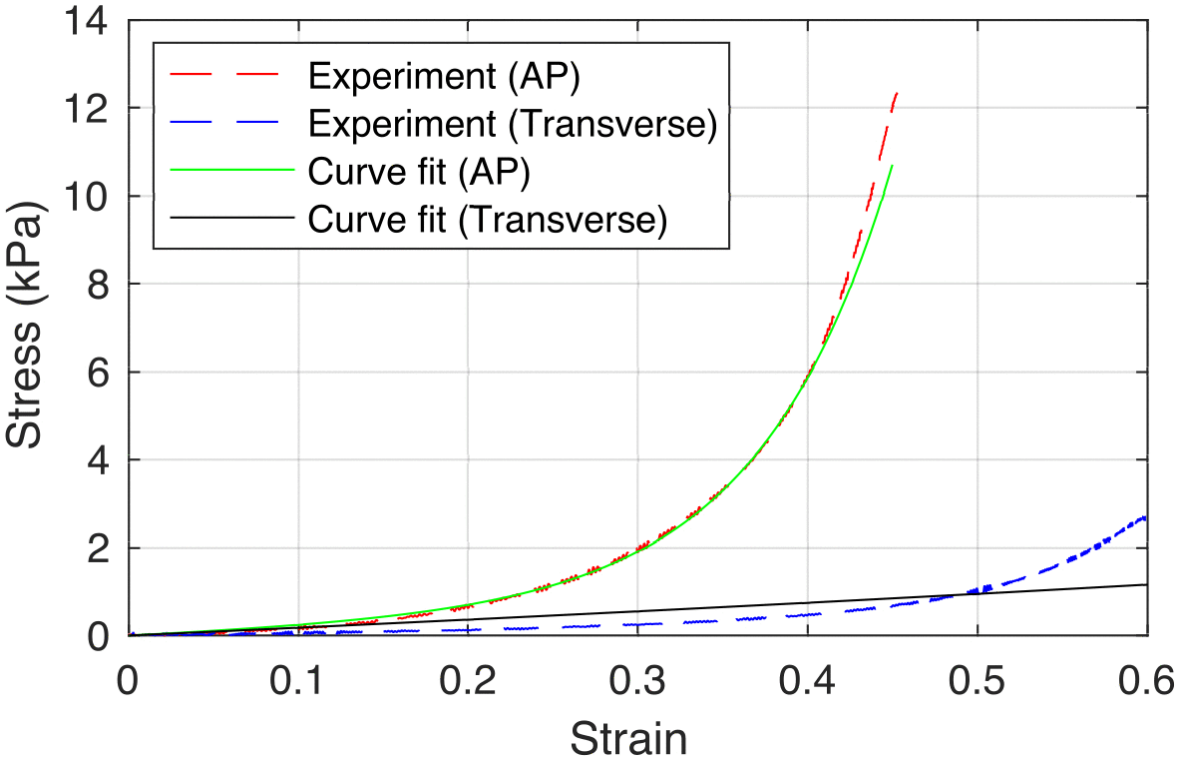
The experimentally measured and curve fitted stress-strain relationship from biaxial tensile testing of a human vocal fold cover layer along the anterior-posterior (AP) and transverse directions.

To estimate the material constants of the anisotropic vocal fold properties, we fitted Eq. (4) to the stress-strain curve obtained from biaxial tensile tests of a human vocal fold cover layer along the anterior-posterior and transverse directions [30]. The experimentally measured and curve fitted stress-strain curves are shown in Fig 3. The estimated material constants c=150 Pa, $k_{\text{1}},\;=\;\text{1}\text{2}\text{7}\text{ Pa}$, and $k_{\text{2}}=\text{2}.\text{5}\text{2}\text{ Pa}$ were obtained using the nonlinear least-square solver *lsqnonlin* in MATLAB 2021b (The Mathworks, Inc.).

For simplicity, the passive mechanical properties of other laryngeal muscles were modeled as nearly incompressible isotropic hyperelastic materials following the approach of a previous study [21]:


$$W_{\text{passive}}=c_{\text{1}}\left({\overset{―}{I}}_{\text{1}}-\text{3}\right)+c_{\text{2}}{\left({\overset{―}{I}}_{\text{1}}-\text{3}\right)}^{\text{2}}+c_{\text{3}}{\left({\overset{―}{I}}_{\text{1}}-\text{3}\right)}^{\text{3}}+\frac{\kappa }{\text{2}}(J-\text{1}{)}^{\text{2}},$$ (5)

where the material constants were set as $c_{\text{1}}$= 1230 Pa, $c_{\text{2}}$= 6514 Pa, $c_{\text{3}}$= 30677 Pa. These values were estimated by curve fitting the experimentally measured stress–strain curve as reported in [31], which was obtained in a tensile testing experiment using excised human larynges. The paraglottic space tissues between the thyroarytenoid muscle and thyroid cartilage was modeled as isotropic hyperelastic materials using a simple neo-Hookean model:


$$W_{\text{passive}}=c\left({\overset{―}{I}}_{\text{1}}-\text{3}\right)+\frac{\kappa }{\text{2}}(J-\text{1}{)}^{\text{2}},$$ (6)

with the material constant c=50 Pa and $\kappa =\text{1}.\text{0}\times {\text{1}\text{0}}^{\text{4}}\text{ Pa}.$

The active component of the strain energy function was defined following the approach of previous studies [19–21]:


$$W_{active}=\left\{ \;\;\;\;\begin{array}{c} \frac{\text{1}}{\text{3}}\alpha \sigma_{max}\left[\text{4}{\left(\text{1}-\frac{\lambda }{\lambda_{\text{ofl}}}\right)}^{\text{2}}-\text{3}\mathrm{\backslash rightleft}(\text{1}-\frac{\lambda }{\lambda_{\text{ofl}}}\right),\text{0}.\text{5}\leq \lambda /\lambda_{\text{ofl}}\leq \text{1}.\text{4}\\ \text{0},\;otherwise \end{array}\;\right.\;,$$ (7)

where α is the muscle activation level, varying from 0 (at rest) to 1 (maximum activation), $\sigma_{max}$ is the maximum active stress value, and λ is the stretch along the muscle fiber direction ${𝐚}_{\text{0}}$, calculated as $\lambda =\sqrt{{𝐚}_{\text{0}}\cdot 𝐂{𝐚}_{\text{0}}}$. The optimal fiber stretch was set as $\lambda_{\text{ofl}}=\text{1}.\text{4}$. The maximum active stress of the TA, LCA, and IA muscles were assumed to have the same activation strength ($\sigma_{max}=\text{1}.\text{0}\text{5}\times {\text{1}\text{0}}^{\text{5}}\text{ Pa}$) [18], whereas the CT muscle was set to $\sigma_{max}=\text{1}.\text{7}\text{5}\times {\text{1}\text{0}}^{\text{5}}\text{ Pa}$ as in [19].

While the laryngeal muscles often consist of many fiber bundles, in this study each muscle was modeled as one homogeneous muscle bundle with an effective fiber orientation angle, similar to previous modeling studies [18–26]. For muscles other than the TA muscle, the fiber direction **a**_0_ was determined based on previous anatomic studies [32] and modeling studies [18–21]. For the TA muscle, the focus of this study, the fiber direction was measured from the MRI-reconstructed geometry. Because of the difficulty in identifying individual fiber bundles in MRI images, the TA muscle fiber direction was measured by drawing a straight line along the length of the TA connecting the two points of attachment of the TA muscle to the thyroid and arytenoid cartilages. Initially, this was measured from a superior view. However, it was later realized that due to the finite size of the TA muscle, such measured fiber direction varied depending on the location within the TA muscle, as further described below. As a result, a range of TA muscle fiber orientation was identified based on the MRI images from this study and previous studies. This range was then used in a parametric simulation to investigate the effect of TA muscle fiber orientation on the degree of inferior medial bulging and medial surface vertical thickness.

Since the cartilages are significantly stiffer than the vocal folds and muscles and are expected to exhibit only small deformations, they were simulated in this study as a linear elastic material with a Young’s modulus of 1 GPa and a Poisson’s ratio of 0.47, similar to previous studies [21].

**Fig 4:**
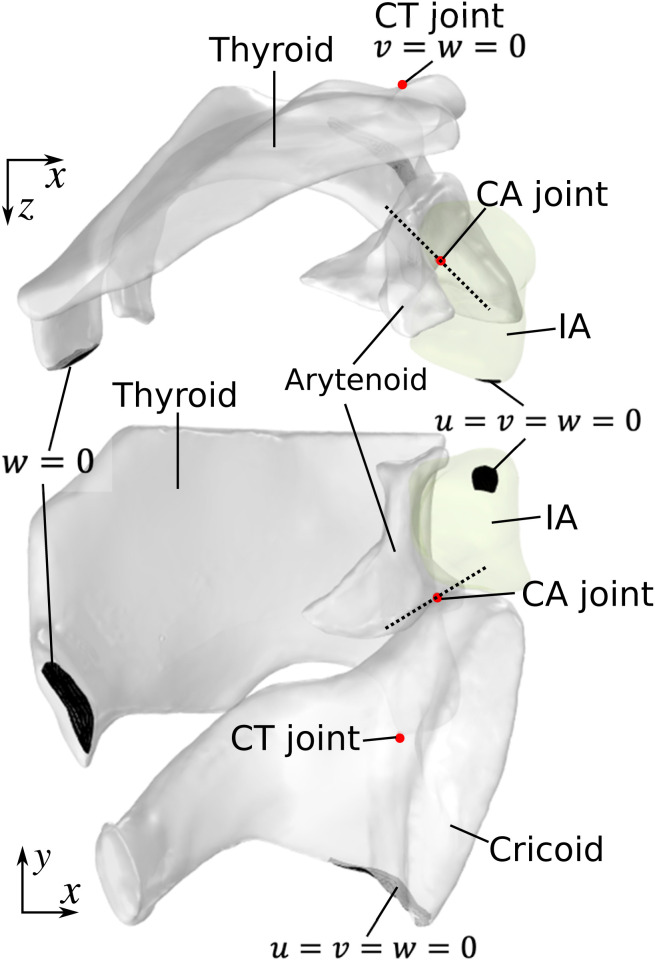
Boundary conditions for the laryngeal cartilages and the interarytenoid (IA) muscle for the male larynx. Same boundary conditions were also applied to the female larynx. Cricothyroid (CT) and cricoarytenoid (CA) joint positions are indicated in red.

The boundary conditions for the computational model are shown in Fig 4. Because only half of the larynx was modeled, the anterior-medial surface of the hemi-thyroid cartilage where it was connected to the other half of the thyroid cartilage was constrained in the left-right direction but free to move in the other directions. Similarly, the medial surface of the IA muscle and the bottom of the cricoid cartilage were also fixed. Within the cricothyroid joint, the thyroid cartilage was allowed to rotate about the z-axis (left-right direction) and translate along the forward-backward direction. As in previous modeling studies [18,20,24], the arytenoid cartilage was allowed to slide along and rotate about the long axis of the cricoarytenoid facet (dotted lines in Fig 4) within the cricoarytenoid joint, based on findings from previous anatomic studies [2].

The computational model was set up and solved using finite element software COMSOL ver. 6.1 (COMSOL inc.). The laryngeal geometry was discretized with a total number of 50,980 quadratic elements. For each muscle, the muscle activation level was increased exponentially from 0.01 to 1, and the equations were solved iteratively until equilibrium condition was achieved for each activation level. The required simulation time depended on the specific muscle activation conditions. Simulation of a single muscle activation typically took about 30 minutes to 2 hours on a DELL Precision Tower with an Intel Xeon Gold 6128 (3.40 GHz) processor and 64 GB of RAM.

### Model validation

**Fig 5:**
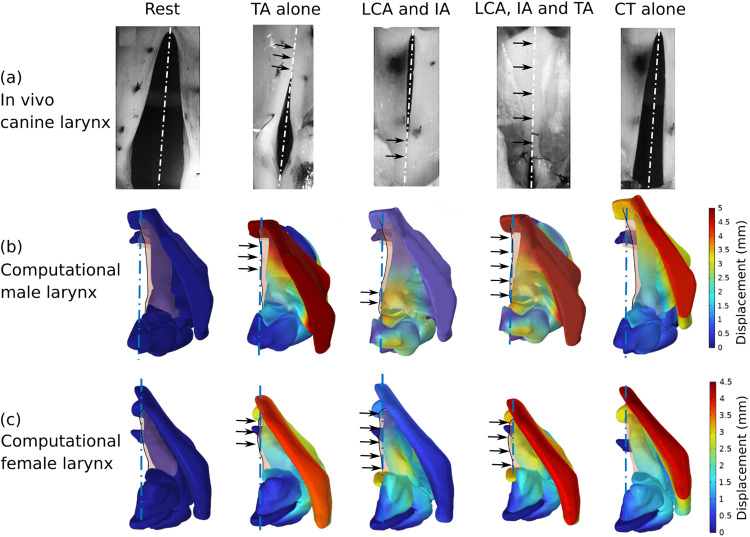
Comparison of glottal configuration under different laryngeal muscle activation conditions from a superior view between (a) in vivo canine larynx [34] and computational simulation in this study of (b) the male and (c) female larynx. The arrows indicate the portion of the glottis that is closed by muscle activation. TA: thyroarytenoid muscle; CT: cricothyroid muscle; LCA: lateral cricoarytenoid muscle; IA: interarytenoid muscle.

Direct validation of our computational model in humans is difficult because it is almost impossible to stimulate individual laryngeal muscles in live humans and observe its impact on voice production, due to the limited access to the larynx and the invasive nature of such experiments. In this study, the computational model was instead qualitatively validated against observations from an in vivo canine experiment [33]. While there are some anatomical differences between the canine and human larynges, previous studies have shown that the canine larynx exhibits similar vibration patterns as humans [e.g., 34]. Fig 5 compares the superior view of the glottal configuration under different conditions of laryngeal muscle activation between the experimental observation and predictions from our simulations. The agreement is reasonably good between experiment and simulation, at least in predicting the general posture changes such as vocal fold adduction, elongation, and shortening. In particular, both the simulation and experiment showed that LCA/IA activation was able to close the posterior glottis but often left a small gap in the anterior glottis, and complete glottal closure required coordinated activation of both the TA and LCA/IA muscles.

### Data analysis

For each muscle activation condition, the changes in the medial surface shape of the vocal fold were visually examined. Because the vocal fold medial surface contour varies smoothly from the superior surface to the inferior surface, it is difficult to identify the lower and upper margines of the medial surface and quantify the medial surface vertical thickness. In previous studies [7,35,36], an effective vertical thickness is often calculated as the vertical span of the medial surface contour within a threshold distance from the most medial point. With a properly set threshold distance, this method quantifies the vertical span of the medial surface that forms the most constricted part of the glottis. In this study, the same method described in [36] was used to calculate the effective vertical thickness, with the threshold value set to 0.4 mm, which provided a consistent vertical thickness in the two larynges used in this study. In general, with inferior medial bulging (i.e., the inferior portion of the medial surface bulges medially more than the superior portion of the medial surface; Fig 1), the medial surface would form a more rectangular or less convergent glottis in the vertical plane, which would increase the effective vertical thickness of the medial surface. In contrast, superior medial bulging (i.e., the superior portion of the medial surface bulges medially more than the inferior portion of the medial surface) would form a more convergent glottis in the vertical plane, thus decreasing the effective vertical thickness of the medial surface.

## Results

### Exploring factors contributing to inferior medial bulging with TA muscle activation

**Fig 6:**
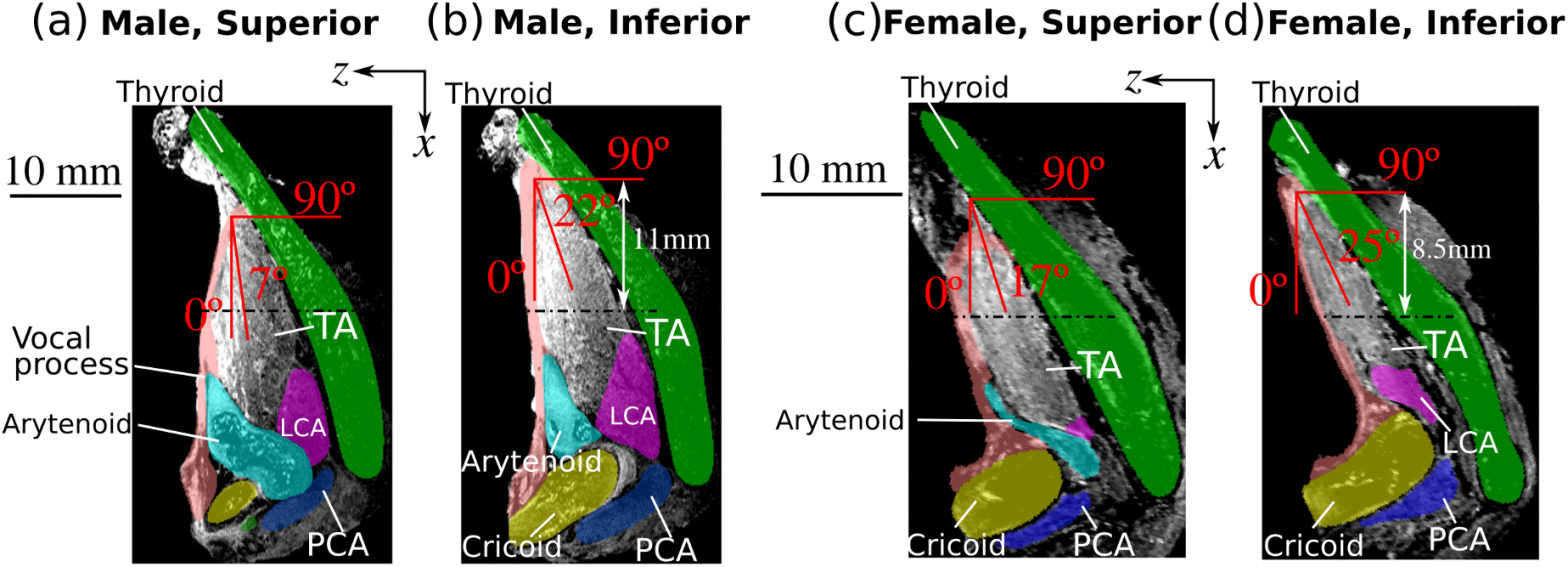
The superior (a, c) and inferior portions (b, d) of the thyroarytenoid muscle exhibit slight differences in muscle orientation in the male larynx (a, b) and the female larynx (c, d). The red line in each panel shows the average TA muscle orientation. The dash-dot lines indicate the mid-section of the vocal fold in the anterior-posterior direction.

Initially, the TA muscle fiber orientation was determined from a superior view, which resulted in a TA muscle fiber angle that was 7º and 17º from the anterior-posterior direction in the male and female larynx, respectively (Fig 6a and 6c). However, activation of the TA muscle in the computational model of the male larynx did not produce noticeable inferior medial bulging of the vocal fold as reported in previous experimental studies [14,15]. In an effort to increase the degree of inferior medial bulging, we systematically adjusted the TA muscle’s attachment area to the thyroid cartilage, the attachment between the paraglottic space (PGS) and the vocal fold, and some constitutive model constants. However, these adjustments did not produce noticeable changes in the degree of inferior medial bulging. We also altered the constraint within the cricoarytenoid (CA) joint by adjusting the angle of the predefined axis of arytenoid movement, which affected the degree of vocal fold adduction under lateral cricoarytenoid (LCA) and interarytenoid (IA) muscle activation but not much in the degree of inferior medial bulging.

**Fig 7:**
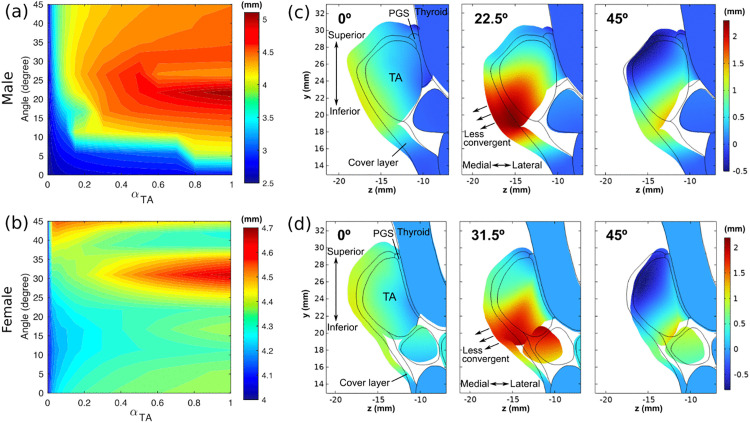
Effective vertical thickness in the mid-coronal plane for different thyroarytenoid (TA) muscle fiber angles and TA activation levels for the male (a) and female (b) larynges. Mid-coronal cross-sections of the larynx are plotted for the male (c) and female larynx (d), respectively, with the medial-lateral displacement coded in color. Black lines indicate the initial geometry.

In contrast, noticeable inferior medial bulging was observed in the computational model of the female larynx. This observation led us to examine the orientation of the TA muscle fibers more closely, as we observed a substantial difference in TA muscle orientation between the male and female larynges (Fig 6). Defining the muscle fiber angle to be 0º along the anterior-posterior direction and 90º along the medial-lateral direction, we found that the superior portion of the male TA muscle had an overall fiber angle of 7º (Fig 6a), which was used in our initial simulations, whereas the fiber angle was approximately 22º at the inferior portion (Fig 6b). In the female larynx, the superior TA muscle exhibited a 17º angle and the inferior part of the TA muscle had an angle of 25º (Fig 6c and 6d). This variation in TA muscle fiber angle from the superior to the inferior vocal fold was also observed in a recent MRI measurement [37]. To examine the effects of TA muscle fiber angle on the degree of inferior medial bulging and medial surface vertical thickness, a parametric simulation was performed by systematically varying the TA muscle fiber angle while keeping the other aspects of the model unchanged. Based on measurements from our MRI images and findings from previous imaging data of the vocal folds [27,37] and laryngeal cartilages [28], the TA muscle fiber angle in the muscle constitutive model was varied from 0º to 45º in steps of 4.5º. The results are shown in Fig 7.

Fig 7a and 7b show the changes in the effective vertical thickness with TA muscle activation for different TA muscle fiber angles in the male and female larynx, respectively. The effective thickness was calculated at the mid-coronal plane of the vocal fold in each larynx (dash-dot line in Fig 6). In the male larynx, the vertical thickness reached its maximum at a fiber angle of 22.5º, whereas in the female larynx, the maximum occurred at 31.5º. For a fiber angle of 22.5º in the male larynx, the vertical thickness increased from 2.5 mm to 5.1 mm as the TA muscle activation increased from 0% ($\alpha_{\text{TA}}=\text{0}$) to 100% ($\alpha_{\text{TA}}=\text{1}$), a range comparable to that observed in in vivo canine larynx experiments [36]. In contrast, the minimum vertical thickness in the female larynx was 4 mm, with only a maximum increase of 0.7 mm under full TA muscle activation. This indicates the female larynx had a relatively larger initial vertical thickness but a more limited range of thickness variation.

Fig 7c and 7d show the mid-coronal cross-section of the resting (solid lines) and deformed larynx for three fiber angles, with the medial-lateral displacement color coded (raw data available in S4 Data). Strong inferior medial bulging can be observed at an optimal fiber angle of 22.5º and 31.5º in the male and female larynx, respectively, in which inferior medial bulging can be clearly observed: the medial surface shape became more rectangular and less convergent. For smaller fiber angles (0º), the medial displacement did not vary much along the medial surface, thus having only small effect on the medial surface shape. For larger fiber angles (45º), the inferior medial bulging was still noticeable, but the vocal fold was also slightly abducted.

**Fig 8:**
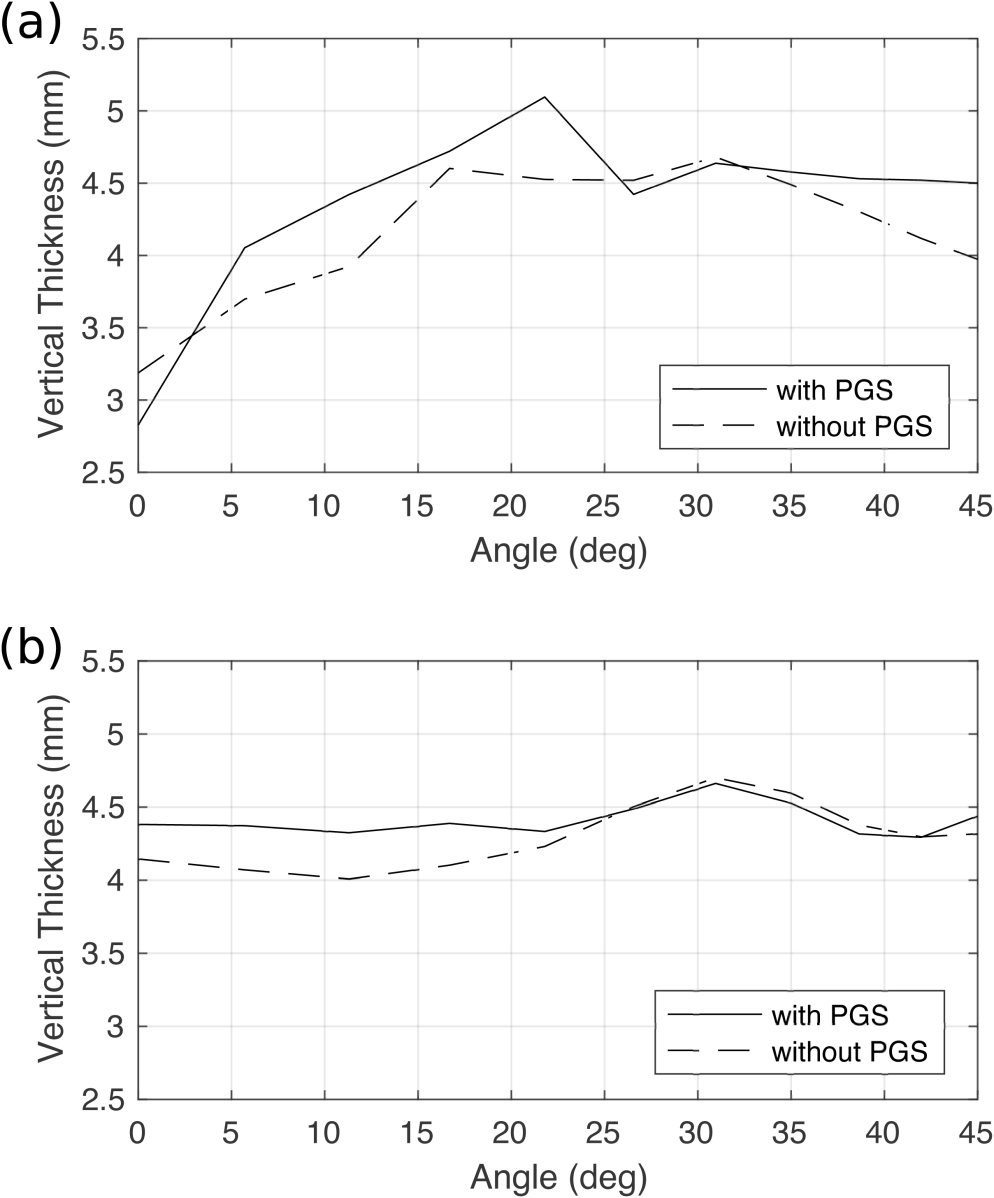
Changes of vertical thickness under full TA activation as a function of the TA muscle fiber angle with and without the paraglottic space (PGS) tissue in the male (a) and female (b) larynx.

It should be noted that in the current model, a large portion of the posterior TA muscle was connected to the thyroid cartilage through the paraglottic space tissues. To examine the effects of the paraglottic space tissues on the vertical thickness, we removed the paraglottic space tissues from the model and repeated the simulations. The results (Fig 8) showed that the fiber angle at which the maximum vertical thickness occurred remained largely the same in both the male and female larynges, indicating that the vocal fold attachment to the thyroid cartilage in the paraglottic space has only a minor influence on the degree of inferior medial bulging.

**Fig 9:**
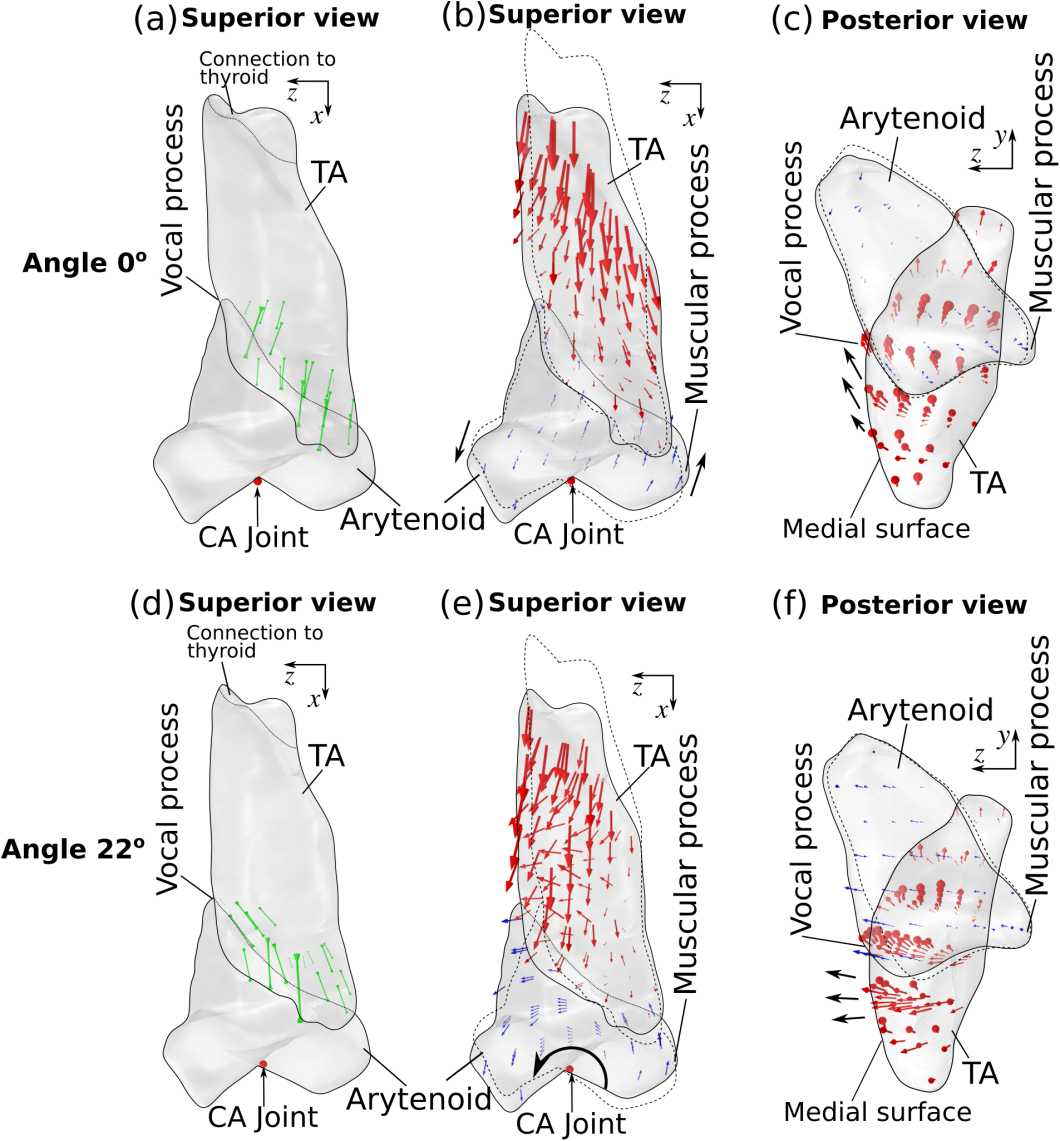
The effect of TA muscle fiber angle on the stress applied to the arytenoid cartilage by the TA muscle (green arrows in panels a and d), the displacement of the TA muscle (red arrows in panels b, c, e, and f) and arytenoid cartilage (blue arrows in panels b, c, e, and f) under full activation of the TA muscle. The top panels are for conditions with a TA muscle fiber angle of 0º, whereas the bottom panels are for conditions with a TA muscle fiber angle of 22.5º. The dotted lines indicate the initial geometry. The black arrows indicate the characteristic motion of arytenoid cartilage and vocal fold medial surface.

To illustrate why the TA muscle fiber angle had a large impact on inferior medial bulging, Fig 9 shows the principal stress applied to the arytenoid cartilage by the TA muscle and the displacement of the TA muscle and arytenoid cartilage in the male larynx for a TA muscle fiber angle of 0º and 22.5º. For a TA fiber angle of 0º, the force acting on the arytenoid cartilage by the TA muscle was largely along the anterior-posterior direction (vertical direction in Fig 9a; S1 Video), whereas for the fiber angle of 22.5º the force had a noticeable medial component (Fig 9d; S2 Video). This medial force induced a rotation of the arytenoid cartilage in the horizontal plane (Fig 9e) and noticeable medial movement of both the vocal process (blue arrows in Fig 9e) and the inferior medial surface (red arrows in Fig 9f). As this medial displacement was induced by the movement of the arytenoid cartilage, its magnitude decreased superiorly (top in Fig 9f), resulting in a strong inferior medial bulging of the TA muscle (Fig 7c). In contrast, for the fiber angle of 0º, the TA muscle force, lacking a strong medial component, was unable to produce a rotation of the arytenoid cartilage or a strong medial displacement of the vocal process (Fig 9b). As a result, the medial displacement of the arytenoid cartilage was much reduced (compare Fig 9b and 9e), and the displacement vectors of the arytenoid cartilage indicated a slight upward motion of the vocal process (Fig 9c). Similarly, the TA muscle exhibited a dominantly superior movement, with a much smaller medial movement (Fig 9c). The medial movement was also more uniform along the medial surface (Fig 9c), resulting in almost negligible inferior medial bulging. These results suggest that fiber orientation with a considerable medial component is essential to inducing inferior medial bulging.

### Effects of LCA and IA muscle activation on medial shape

**Fig 10:**
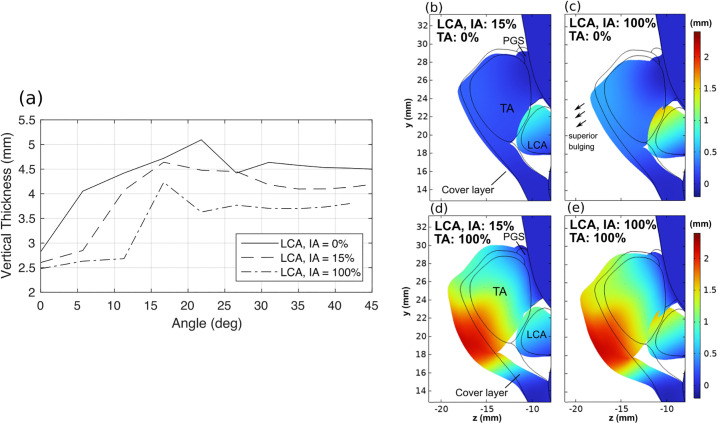
(a) Changes in the medial surface vertical thickness at full TA muscle activation as a function of the TA muscle fiber angle for different levels of LCA/IA activation in the male larynx. (b-e) Mid-coronal cross-sectional shape of the male larynx under different conditions of TA/LCA/IA activations for a TA muscle fiber angle of 22.5º, with the vocal fold medial displacement color coded. Black lines indicate the initial geometry. TA: thyroarytenoid muscle; LCA: lateral cricoarytenoid muscle; IA: interarytenoid muscle.

The previous section shows that a medial movement of the vocal process is essential to inducing the inferior medial bulging of the vocal folds. However, the LCA muscle, which attaches to the arytenoid cartilage and cricoid cartilage, also medializes the vocal process [33] but has been shown to induce mainly superior medial bulging [7,15], which reduces vocal fold vertical thickness. To clarify the mechanism underlying this difference, we simulated activation of the LCA and IA muscles, both of which contribute to medialization of the posterior part of the vocal folds, with and without simultaneous TA activation. Fig 10a shows the medial surface vertical thickness as a function of the TA muscle fiber angle at 100% TA muscle activation and varying degrees of LCA/IA activation. The vertical thickness decreased with increasing LCA and IA muscle activation, although a significant increase in the vertical thickness was still observed when the TA muscle fiber angle changed from 0º to 17º. This suggests that the effect of TA muscle fiber angle on the inferior medial bulging remains dominant even with simultaneous LCA and IA muscle activation. The mid-coronal cross-sectional view of the vocal folds (Fig 10c) shows that LCA/IA muscle activation caused the superior portion of the medial surface to bulge medially more than the inferior portion of the medial surface. As a result, with LCA/IA activation the medial surface was more convergent compared to that without LCA/IA activation, as shown in Fig 10c, which reduces the medial surface vertical thickness, consistent with experimental observation [15]. With simultaneous TA, LCA, and IA muscle activation (Fig 10e), the vocal fold was more medialized compared to the case with dominant TA muscle activation (Fig 10d). However, the vertical thickness was slightly smaller in Fig 10e due to the superior medial bulging associated with LCA/IA muscle activation.

**Fig 11:**
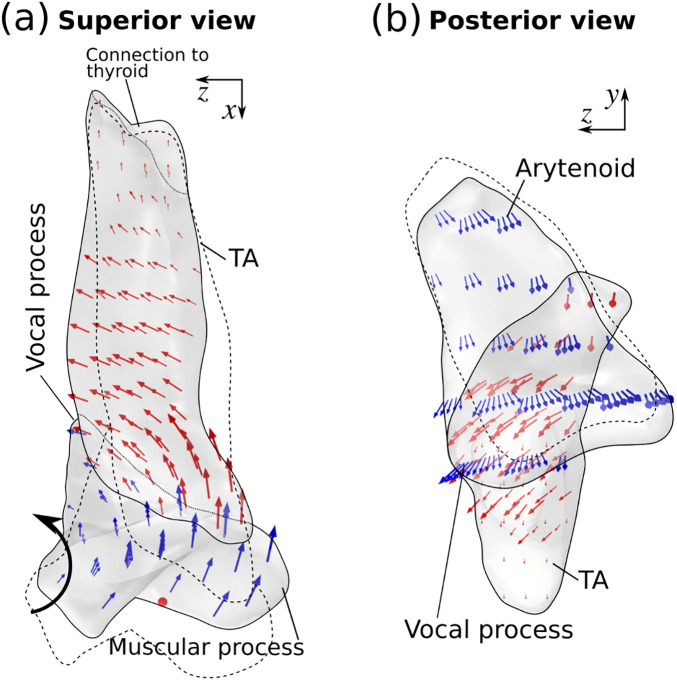
Displacement vectors within the arytenoid cartilage (blue arrows) and thyroarytenoid muscle (red arrows) under full activation of the lateral cricoarytenoid (LCA) muscle in the superior view (a) and posterior view (b). The TA muscle was not activated. The dotted lines indicate the initial geometry. The black arrow indicates the rocking motion of arytenoid cartilage.

Fig 11 shows the displacement vectors of the TA muscle and arytenoid cartilage under activation of the LCA muscle alone (also S3 Video). The LCA muscle applied an anterior, medial, and downward force to the muscular process of the arytenoid cartilage. This induced a rocking motion of the arytenoid cartilage about the long axis of the cricoarytenoid facet in the vertical plane (see also [20]), which medialized the vocal process and the posterior vocal fold. This is different from the arytenoid rotation in the horizonal plane induced by TA muscle activation. Because of this arytenoid rocking motion, while the vocal process was medialized, the medial movement at the superior part of the TA muscle was larger than that at the inferior part of the TA muscle (Fig 11b), resulting in an overall superior medial bulging of the medial surface. This suggests that to realize inferior medial bulging and increase medial surface vertical thickness, the vocal process needs to be medialized through a horizontal rotation of the arytenoid cartilage rather than a rocking rotation in the vertical plane.

### Vertical thickness reduction with CT muscle activation

**Fig 12:**
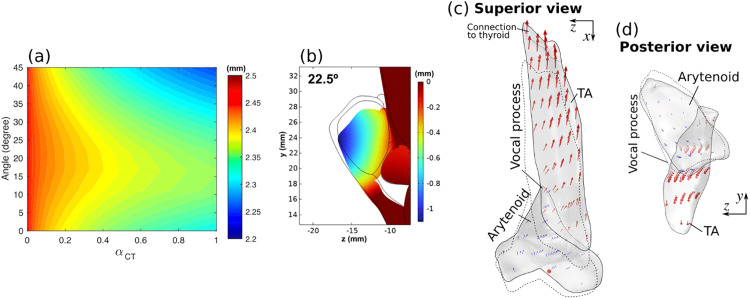
Changes in the vertical thickness, mid-coronal cross-section, and displacement vectors for the case with cricothyroid (CT) muscle activation alone. (a) Changes in the effective vertical thickness under different TA muscle fiber angles and CT activation levels $\alpha_{𝐂𝐓}$. (b) Mid-coronal cross-section of the vocal fold with and without CT activation, with the thin lines indicating the initial geometry and the medial displacement color coded. Displacement vectors in the arytenoid cartilage (blue arrows) and TA muscle (red arrows) at $\alpha_{𝐂𝐓}=1$ are plotted from a superior view (c) and a posterior view (d), with dotted lines indicating the initial geometry.

Previous experimental studies showed that in general the vertical thickness decreases slightly with the activation of the cricothyroid (CT) muscle (e.g., [14]), although the opposite was also observed [38]. In this study, we also examined the effect of CT muscle activation alone on the medial surface vertical thickness. When the CT muscle was fully active, the vocal fold was elongated by approximately 18%, which is consistent with observations in previous studies [39,40]. The vertical thickness as a function of the TA muscle fiber angle and CT muscle activation level is shown in Fig 12a. Increasing CT activation decreased the vertical thickness, but the effect size (approx. 10% decrease or 0.2 mm reduction) was much smaller than the effect size of TA muscle activation (approx. 104% increase). Interestingly, the decrease in vertical thickness was the smallest at an intermediate TA muscle fiber angle. This is because CT muscle activation also modified the medial surface shape to be less convergent, as shown in Fig 12b. This medial surface shape change was caused by a slight horizontal rotation of the arytenoid cartilage, similar to that observed under TA muscle activation but in the opposite direction and with a much-reduced magnitude (Fig 12c). This led to a lateral movement of the TA muscle that was slightly stronger at the superior portion of the TA muscle than the inferior portion (Fig 12d). While this medial surface shape change would have increased the vertical thickness, this effect was outweighed in this case by the reduction in the cross-sectional area due to vocal fold elongation, resulting in an overall effect of decreasing thickness. It is possible that at some conditions the effect of medial surface shape change may outweigh the effect of cross-sectional reduction, which may explain the observation in [38].

## Discussion

Each voice is unique, with some speakers modulating their voice quality more easily than others. Our results suggest that this individual difference in modulating voice quality may be at least partially related to individual difference in TA muscle fiber orientation, which critically determines the ability to regulate vocal fold vertical thickness, an important parameter in regulating voice quality. In our study, the maximum range of thickness control occurred at an optimal range of TA muscle fiber angle between 22–32°. Our results further showed that the key to inferior medial bulging and thickness control is the ability to induce a horizontal, medial rotation of the arytenoid cartilage, which resulted in maximal medial displacement of the vocal process without simultaneous medial displacement of the superior medial surface. In contrast, a rocking motion of the arytenoid in the vertical plane, as induced by LCA/IA muscles, had an opposite and much smaller effect on the medial surface shape and vertical thickness.

At these optimal TA muscle fiber angles, our computational model showed significant inferior medial bulging and increased vertical thickness with TA muscle activation that are consistent with previous experimental observations [14,15]. This change in the vertical thickness was substantially larger than those reported in previous computational models, e.g., [19,20,26], probably due to the differences in the specific fiber angles. The previous study [7] suggested that a medial surface shape change in the order of 0.5 mm significantly altered the voice quality. Thus, changes in vertical thickness as observed in the current study (as large as 2.5 mm) likely have a large impact on voice quality.

The importance of the TA muscle fiber angle to the control of vocal fold vertical thickness and voice quality points to the need in future studies to quantify TA muscle fiber angle distribution in a large number of human subjects. While there are currently no data available on the individual variability in TA muscle fiber orientation in humans, Hunter and Titze [28] reported large individual differences in the size and relative positions of the thyroid, cricoid, and arytenoid cartilages. Since the TA muscle is attached to the thyroid and arytenoid cartilages, this large individual difference in the size and relative positions of laryngeal cartilages suggests likely a large individual difference in the overall TA muscle orientation. The effective orientation of the individual TA muscle fiber also depends on the geometry and size of the TA muscle. Thus, potential individual differences in the effective TA muscle fiber angle, as considered in this study, may also result from the large individual differences in TA muscle geometry and size, as demonstrated in Fig 6 and findings from previous imaging studies (e.g., [27,37]). While voice quality also depends on many factors in the respiratory and articulatory subsystems [1,2], at the laryngeal level, speakers who can produce large changes in the vertical thickness will be better able to modulate their voice from a breathy to pressed voice, whereas those with limited vertical thickness variation will likely speak with a relatively limited modulation in voice quality [4]. Thus, our results suggest that individuals with a TA fiber angle close to the optimal angles of this study may be able to exert greater control over their voice source, which may allow them to more easily modulate voice quality, such as in singing, acting, or mimicking others’ voice. This hypothesis needs to be tested in humans. It is possible that quantifying the TA muscle fiber orientation in humans may provide important information on an individual’s potential vocal capability or vocal health risks, which has important applications in both clinical voice care and vocal pedagogy. Future research should focus on developing non-invasive methods for in vivo quantification of the TA muscle fiber orientation.

A limitation of this study is that the observed muscle bulging was a combined result of volume conservation of the muscle (muscle shortening leads to cross-sectional expansion; Fig 7) and arytenoid movement. There are other mechanisms (e.g., surge in intramuscular pressure due to muscle activation and the restraining effects of fascia and connective tissues) that might contribute to additional muscle bulging and were not considered in this study. Another limitation is that the TA muscle was modeled as one homogeneous fiber bundle with a single effective fiber orientation. Due to the finite size of the laryngeal muscles, each muscle consists of many fiber bundles with slightly different fiber orientations. For example, the TA muscle is often divided into a medial and a lateral bundle, with the lateral bundle likely having a large fiber orientation with respect to the anterior-posterior direction [2,18]. The orientation of each muscle fiber may also vary along its length. Such inhomogeneous fiber orientation may lead to nonuniform muscle bulging [41]. While these simplifications (neglecting micro-structural mechanisms and modeling the TA muscle as a single effective fiber orientation) follow previous modeling studies of laryngeal muscle activation, their impact on the findings of the present study needs to be investigated in the future when detailed anatomic data become available. Finally, only two larynges were considered in this study. Considering the large individual anatomic variations [27,28,37], this study needs to be repeated in a large number of larynges from a wide age range for both sexes. A systematic sensitivity study is required to evaluate the impact of the model setup, including the mechanical properties of the larynx and boundary conditions, on the findings of this study.

## Supporting information

S1 Video Movement and displacement vectors of TA muscle and arytenoid cartilage with a fiber angle of 0º when the TA muscle was activated from 0 to 100%. (MP4)

S2 Video Movement and displacement vectors of TA muscle and arytenoid cartilage with a fiber angle of 22.5º when the TA muscle was activated from 0 to 100%. (MP4)

S3 Video Movement and displacement vectors of TA muscle and arytenoid cartilage with a fiber angle of 22.5º when the LCA muscle was activated from 0 to 100%. (MP4)

S4 Data Medial surface data at different fiber angles and stimulation conditions for the male and female larynx. (ZIP)
